# Inter-organizational social capital of firms in developing economies and industry 4.0 readiness: the role of innovative capability and absorptive capacity

**DOI:** 10.1007/s11846-022-00539-3

**Published:** 2022-03-26

**Authors:** Najam ul zia, Ladislav Burita, Yumei Yang

**Affiliations:** 1 Tomas Bata University in Zlin, Mostní 5139, 760 01 Zlín, Czechia; 2 University of Defense in Brno, Kounicova 65, 662 10 Brno, Czechia; 3 Bournemouth University, 19 Christchurch Road, Bournemouth, United Kingdom

**Keywords:** Industry 4.0, Interorganizational social capital, Innovative capability, Absorptive capacity, Developing economy

## Abstract

The globalization of markets provides opportunities for firms to collaborate on various activities. These collaborations are the resource of social capital for firms to achieve sustainable competitiveness. This study aims to investigate how social capital (i.e., structural, relational, and cognitive social capital) between firms in a developing economy and developed economy strengthens their innovation capability and enhance their industry 4.0 readiness. Using Smart PLS-SEM to analyse the data collected from 320 managers representing 81 manufacturing firms in Pakistan, we found that social capital is positively associated with industry 4.0 readiness, and innovative capability mediates this relationship. The study contributes to the existing knowledge of understanding industry 4.0 readiness and provides useful insights for firms in developing economy to improves their innovation capability during the industry 4.0 era. This study likewise reveals the significance of three dimensions of social capital, which can facilitate to bring in digital knowledge from developed economies to developed economies to get ready for the fourth industrial revolution.

**Supplementary Information:**

The online version contains supplementary material available at 10.1007/s11846-022-00539-3.

## Introduction

We are at the cusp of the fourth industrial revolution which significantly influence the organizational operations (Ghobakhloo, 2020; Reinhard et al., 2016). The fourth industrial revolution represents the digital transformation in existing businesses and changing the manual working methodologies with digital computer structures (Iansiti & Lakhani, 2014). It also refers to a value creation network that increases intelligence of products and systems through their intra-company and inter-company integrations (Scheneider, 2018). The potential to embrace industry 4.0 technologies such as cyber physical systems, internet of things and big data is defined as industry 4.0 readiness (Geissbauer et al., 2016).The organizations use cyber physical systems and IOTs to automate their production systems. However, companies face challenges to embed IOTs in business processes (Chen et al., 2014; I. Lee & Lee, 2015; Qian & Wang, 2012). Hence, it is important for companies to be ready for Industry 4.0. Many companies have desire to be at the maturity level of Industry 4.0 readiness, but they demonstrate little digital maturity and lack of plan for Industry 4.0 implementation (Antonsson, 2017). It is crucial to investigate and identify the factors potentially enhancing Industry 4.0 readiness to reap the maximum value out of it.

Digital economy has already created new strategic options and gains for industrialized economies. However, firms in developing economies still rely on labour intensive, less specialist, and low technological skills to service low-cost market segments (Malik & Kotabe, 2009). Less emphasis on research and development and lack of technological capabilities pushes firms in developing economies to rely on industrialized economies to purchase new technologies (Shamim et al., 2019; Awan et al., 2021; Shamim, Zeng, Khan, et al., 2019). This phenomenon reflects very low level of Industry 4.0 readiness among firms in developing economies. Less developed economies face the issue of institutional voids, where organizations receive little or none support from home institutions for knowledge and innovations (Khan et al., 2019). In this situation, external sources of knowledge such as network of suppliers, partners, and customers, become more important. Particularly, firms in developing economies having networks with firms in digitally advance economies such as supplier, customer, or partner network can gain knowledge from those firms to enhance their innovative capability (Khan et al., 2019), which leads to Industry 4.0 readiness. When it comes to extract knowledge, support, and resources from relationship networks, social capital plays an important role at explaining value creation through social interaction (Mazzucchelli, Chierici, Tortora, & Fontana, 2019).

Social capital refers to interpersonal relationship network, which provides resources such as information, trust, and support for value creation (Nahapiet & Ghoshal, 1998). Social capital can enhance the production performance (Serra & Poli, 2015). Dimensions of social capital are structural social capital, relational social capital, and cognitive social capital (Tsai & Ghoshal, 1998). Consistent with Mazzucchelli et al. (2019) we believe that these three dimensions would be associated with the outcomes of industry 4.0. Particularly these dimensions of social capital are positively associated with innovative capability of firms involved in international operations (Sheng & Hartmann, 2019). Innovative capability of firms is one of the desired competencies for Industry 4.0 readiness (Shamim et al., 2016). In the fourth industrial revolution, firms can drive sustainable competitive advantage by utilizing the tangible and intangible resources to enhance their Industry 4.0 readiness. Existing literature is evident that innovative capability of firms facilitates the path towards Industry 4.0 (Agostini and Filippini 2019). Lasi et al. (2014) also argued that high innovative capability is an essential factor for enterprises operating the environment of Industry 4.0. Based on the discussion here, it seems social capital is indirectly linked with industry 4.0 readiness via innovation capability. However, the relationship has never been test. this study fills this gap by empirically examining the association of innovative capability, social capital, and Industry 4.0 readiness.

Firms in developing economies rely on industrialized economies for Industry 4.0 technologies (Shamim, Cang, Yu, et al., 2019) but these firms must have capacity to assimilate the knowledge and transfer it for innovation. This ability to understand, evaluate, assimilate and apply the external knowledge is recognized as absorptive capacity (Cohen & Levinthal, 1989). Absorptive capacity is studied as an important factor influencing firm innovation (Cohen & Levinthal, 1989; Knudsen, 2007). However, there is limited existing research to understand the impact of absorptive capacity on utilizing the firm’s external knowledge from social capital, then how the external knowledge is transformed to improve innovation capability.

To address the gaps we identified above, this study investigates the social capital of the manufacturing firms based in Pakistan, which are running businesses with their business partners in developed and industrialized economies. We chose Pakistan to investigate these issues; particularly Pakistani manufacturing firms who have relationship network in technologically advanced economies and extract resources, knowledge, and support from their networks. Pakistan provides a suitable context of developing economy, and it is facing the issue of institutional voids (Khan et al., 2019). The investigation aims to identify the role of social capital in enhancing Industry 4.0 readiness of the manufacturing firms in developing economies. Furthermore, it examines the mediating role of innovative capability in the relationship of social capital and Industry 4.0 readiness, and the moderation role of absorptive capacity on the linkage between social capital and innovative capability. This study contributes to the literature in multiple ways. First, it adds to the body of knowledge about industry 4.0 readiness in developing economy. This compensates the current literature of industry 4.0 readiness with studies mainly from industrialized economy, where advanced technologies are easily to be adopted. Second, the study moves the focus from investment on emerging technologies to the organizational context in relation to industry 4.0. It shows that organizations should not only focus on technologies, but also need to make use of the networks with external business partners to improve their innovative capabilities and industry 4.0 readiness.

## Theoretical and hypotheses

### Social Capital

The social capital refers to value creation through network of relationship (Chuang, Chen, & Chuang, 2013). Social capital is closely linked with the level to which people share information, and other resources implanted in the network of relationship (Wang & Ho, 2017). It is also possible to induct resources in an organizational structure to get new technology adoption and improvement (Parellada et al., 2011). Social capital plays an important role in organizational innovation and presentation (Sánchez et al., 2015). Social capital theory suggests that sociability is necessary and critical requirement for valued resource. It also proposes that all the relationships between organizational members and outside players are prerequisites for innovation (Shamim et al., 2021; Shamim, Cang, & Yu, 2019), knowledge creation and information sharing (Zhang & Peterson, 2011).

Social capital promotes organizational performance by enabling access to key resources and information (Johnson et al., 2013). Moreover, social capital can influence organizations efficiency through knowledge sharing and innovation (Tsai & Ghoshal, 1998). There are three dimensions of social capital i.e. structural social capital, relational social capital and cognitive social capital (Nahapiet et al., 1998), that are used and explained in this study. The structural social capital explains who will interact for building relations and how these relations will be attained (Chow & Chan, 2008). This dimension comprises of factors like, density, network patterns, hierarchy, and connectivity (Hughes & Perrons, 2011). It refers to the social system properties and network of relations (Nahapiet & Ghoshal, 1998). It is an impersonal configuration of relations between people and units that includes the procedures, precedents, rules and roles which are considered as expressions of this configuration (Uphoff & Wijayaratna, 2000). Structural social capital provides possibilities to access the various parties for transferring and exchanging knowledge, and facilitates to increase the opportunity to exchange (Ansari et al., 2012). It also facilitates the people to contact their peers for the sake of knowledge and expertise (Andrews, 2010).

Relational social capital is the most sentimental component of social capital that describes networks in terms of shared norms, interpersonal trust and connection with other people (Cabrera & Cabrera, 2005). This dimension of social capital directs to the quality and nature of the relationships that can be developed through a history of interactions with each other or to other parties (Lefebvre et al., 2016) and contributes in several behavioural attributes like, obligations, trustworthiness, shared group norms and identification (Davenport & Daellenbach, 2011). Nahapiet and Ghoshal (1998) stated the main aspects of this dimension are obligations and expectations, norms and sanctions, and trust and trustworthiness. The relational of why social capital supports normative behaviour is based on expectations, reciprocity, trust, and obligations (R. Lee & Jones, 2008). Digital environments are created through trust that can be derived through knowledge sharing and transactional behaviour (Ridings et al., 2002).

Finally, the third component is cognitive social capital that includes the values, vision and shared goals of organizational members (Wasko & Faraj, 2005). Cognitive social capital relates to resources providing systems of meaning, interpretations and shared representations among parties (Nahapiet & Ghoshal, 1998). It is a shared code and language which represents the basics of communication (Gooderham, 2007). Nahapiet and Ghoshal (1998) has linked cognitive social capital to shared narratives and shared language, whereas other authors described it through shared culture, vision and shared goals (Inkpen & Tsang, 2005; Tsai & Ghoshal, 1998). These three dimensions of social capital play an important role in developing the innovative capability of any organizations (Ganguly et al., 2019) and this innovative capability ultimately represents industry 4.0 readiness (Sheen & Yang, 2018).

Literature suggests that less developed economies rely on industrialized economies for smart digital technologies (Shamim et al., 2019). Khan et al. (2019) also argued that firms in less developed economies seek knowledge and support from external sources. When it comes to knowledge extraction, social capital is one of the most established tools in this context (Maurer et al., 2011). Social capital influences technological dynamism (García-Villaverde, Rodrigo-Alarcón, Parra-Requena, & Ruiz-Ortega, 2018) and strengthens the application of knowledge for radical innovations (Pérez-Luño, Medina, Lavado, & Rodríguez, 2011). It makes social capital a relevant theoretical lens for this study. Social capital can be discussed at inter-organizational and intra-organizational level (Maurer et al., 2011). This study investigates the social capital of firms in developing economy with foreign firms at inter-organizational level.

### Social capital and Innovation capability

Innovative capability refers to the ability of firm to create new and distinguished products, services, and markets, and improving the existing ones (March, 1991). Innovative capability can enable the organization to drive sustainable competitive advantage (March, 1991). In the environment of Industry 4.0 competitive advantage relies on Industry 4.0 readiness (Shamim et al., 2016), which depends on firm’s innovative capability (Agostini and Filippini 2019; Lasi et al., 2014). Therefore, in this context, it is important to identify the enablers of innovative capability of firm.

Social capital is one of the established predictors of innovativeness (Maurer et al., 2011). Social capital theory proposes that the network of inter-organizational and intra-organizational relationships are important prerequisites for innovation (Zhang & Peterson, 2011). Rost (2011) also argued that strength of inter-organizational ties plays crucial role in the creation of innovation. Structural and relational social capital influence innovative capability of firms involved in international operations such as exports (Sheng & Hartmann, 2019). Structural social capital represents the existence of network ties (Inkpen and Tsang, 2005) which provides greater access and flexibility in exploiting existing knowledge and exploring new knowledge which are the prerequisites of innovativeness (Donate and Pablo, 2105). Access to such knowledge enables additional combinations of knowledge and permits greater innovation (Sheng & Hartmann, 2019, Shamim et al., 2021). Relational social capital represents the trust (Andrews, 2010) and trusting relationships trigger freely exchange of knowledge among network actors. Hence, trust improves firm’s learning ability and drives the creation of broader scope of knowledge for building and reconfiguring the sources of innovations (Sheng & Hartmann, 2019). Relational capital through trust can provide access to divergent ideas (Rowley et al., 2000). Trust inspires the firms to search for diverse knowledge resources and encourages alternative ways of actions which enhances the innovative capability of firms (Sheng & Hartmann, 2019). These three dimensions of social capital play an important role in developing the innovative capability of any organization (Ganguly et al., 2019) and this innovative capability ultimately represents industry 4.0 readiness (Sheen & Yang, 2018). Cognitive social capital represents the shared code, languages, values, and goals and it facilitates the sharing of tacit knowledge (Alguesaui et al., 2010) which is a prominent predictor of innovativeness (Hau et al. 2013). Ganguly et al. (2019) also argues that these three dimensions of social capital play an important role in developing the innovative capability of organizations. Based on these arguments following are the hypotheses:

H1: Structural social capital is positively associated with innovative capability.

H2: Relational social capital is positively associated with innovative capability.

H3: Cognitive social capital is positively associated with innovative capability.

### Social Capital, Innovative capability and Industry 4.0 Readiness

Industry 4.0 is closely related to connecting cybernetic, database or digital world with analogue, the physical and tangible world (Quint et al., 2015). Haddara & Elragal, (2015) describe Industry 4.0 as computerization of the manufacturing industry, where Cyber-Physical Systems (CPS) are recognized as integral part of it, and industry experts consider it as technical drivers of Industry 4.0 (Brettel et al., 2014). In the current digital economy, companies need to implement Industry 4.0 strategy to get competitive advantage and sustainability for a longer run with satisfactory performance (Drath & Horch, 2014). Therefore, companies must be equipped to face this new competitive challenge and show readiness to adjust in new technological paradigm (Lee et al., 2014). The basic step towards implementation of Industry 4.0 is to check the digital readiness of the organization and it starts with understanding the existing strengths and weaknesses before initiating this digital paradigm (Geissbauer et al., 2016). The potential to embrace industry 4.0 technologies such as cyber physical systems, internet of things and big data is referred as industry 4.0 readiness (Geissbauer et al., 2016).

Pacchini, Lucato, Facchini, & Mummolo, (2019) initiated the discussion on a model to evaluate the industry 4.0 readiness and highlighted the scarcity of existing literature on the issue of determining the degree of industry 4.0 readiness. Organizations can use different tools to check their readiness towards industry 4.0 (Rajnai & Kocsis, 2018). These tools are set as a benchmark to assess the effective direction of an organization towards digital transformation. Therefore, a successful adaptation of industry 4.0 can only be planned after performing an assessment of industry 4.0 readiness (Maisiri & van Dyk, 2019). Industry 4.0 readiness can be assessed through different levels such as, in an organization, in a department, or at a national level (Basl, 2017). Industry 4.0 readiness does not only link with advanced technological investments but it includes the details of the availability of skills and organizational strategy (Maisiri & van Dyk, 2019). Industry 4.0 is a digital transformation that is not a sudden change; rather, it appears a gradual change that comprises many stages (Rajnai & Kocsis, 2018). The purpose to evaluate the industry 4.0 readiness is to identify the phase of an organization towards digital transformation. Management must have a clear strategic plan after knowing the current status of the organizational trend towards digitization (Rajnai & Kocsis, 2018). Due to increasing importance of information, different assessment models are evolved, and they use numeric indicators of readiness and bundle these points into thematic groups. The outcomes of these indicators are used to calculate the digital readiness indexes and ultimately assessing the digital readiness of organizations (Rajnai & Kocsis, 2018).

Firms in less developed economies rely on developed economies to import industry 4.0 related technologies and products. On the other hand, firms in developed and industrialized economy offshore some of their production facilities to firms in developing economies such as software solutions (Sinkovics et al., 2019). In this situation firm’s social capital with firms in more digital and developed economies can be source of knowledge extraction (Mazzucchelli et al. 2019). Provision of knowledge, information and other resources related to industry 4.0 can help the firms to develop the skills and capabilities to embrace industry 4.0. Structural social capital of firms that refers to establish personal relationships, can play crucial role in retrieving information from other team members (Tsai & Ghoshal, 1998). Similarly, relational social capital increases the breadth, frequency and depth of relationship that improve the information sharing between team (Mazzucchelli et al., 2019). This digital information can be useful for firms in building Industry 4.0 foundation. Furthermore, sharing vision, language goals and interests can also foster team members to share information, and that is a core theme of cognitive social capital (Chow & Chan, 2008). Knowledge becomes smoother in flow when it is transmitted in common language and vocabulary (Tagliaventi et al., 2010). Therefore, sharing digital vision by industries from developed economies to industries in developing economies can support these firms in embracing Industry 4.0 strategy. Industry 4.0 is a strategic choice, and therefore social capital can influence the strategic choices of firms (Houghton, Smith, & Hood, 2009). Based on logical beliefs and these arguments we assume that all three dimensions of social capital are positively related to industry 4.0 readiness.

H4. Structural social capital is positively associated with industry 4.0 readiness.

H5. Relational social capital is positively associated with industry 4.0 readiness.

H6. Cognitive social capital is positively associated with industry 4.0 readiness.

Innovative capability is also an enabler of industry 4.0 (Shamim et al., 2016). Existing literature is also evident that innovative capability of firms facilitates the path towards Industry 4.0 (Agostini and Filippini 2019). This ability enables firms to create new, distinguished and improved production processes (March, 1991). Innovative capability also enables the firms to drive sustainable competitive advantage (March, 1991). In the environment of Industry 4.0 competitive advantage relies on Industry 4.0 readiness (Shamim et al., 2016), which depends on firm’s innovative capability (Agostini and Filippini 2019; Lasi et al., 2014). Lasi et al. (2014) also argued that high innovative capability is an essential success factors for enterprises operating the environment of Industry 4.0. On the other hand innovative capability of firms, particularly those involved in international operations heavily depend on their social capital (Sheng & Hartmann, 2019). Social capital plays an important role in developing the innovative capability of organizations (Ganguly et al., 2019) and this innovative capability ultimately leads industry 4.0 readiness (Sheen & Yang, 2018). Existing literature is also evident of mediating role of innovative capability in the relationship of social capital and its outcomes (Agyapong et al., 2017). Based on these logical beliefs and arguments, we assume that innovative capability is positively related to industry 4.0 readiness, and innovative capability mediates the relationship of social capital and industry 4.0 readiness. So following are the hypotheses.

H7: Innovative capability is positively associated with industry 4.0 readiness.

H8: Innovative capability mediates the relationship of social capital and industry 4.0 readiness.

### Absorptive Capacity

Absorptive capacity is a firm’s ability to understand, evaluate, assimilate and apply the external knowledge (Cohen & Levinthal, 1989, 1990). Several studies offered an expanded definition of absorptive capacity built upon the concept of Cohen and Levinthal. Grünfeld (2003) states absorptive capacity as a firm’s ability to absorb knowledge from other firms whereas Zahra & George (2002) described it as the ability of any organizations to acquire, absorb, convert, and apply external knowledge. According to Tsai & Ghoshal (1998), absorptive capacity is a firm’s ability to effectively imitate new knowledge. Succinctly, absorptive capacity is a firm’s ability to value, understand, assimilate, absorb, and apply knowledge that is obtained from external sources.

Existing studies found that absorptive capacity is an important factor contributing towards the process of knowledge transfer (Soh & Roberts, 2005). The knowledge searching, processing and integrating activities are the key practices for innovation (Yu, 2013) and this knowledge is entailed to enhance their innovative capabilities. Based on the existing literature about absorptive capability, the knowledge reservoir of a firm increases the ability of a firm to value, acquire, assimilate and apply new knowledge ( Cohen & Levinthal, 1990). Research implies that greater absorptive capacity facilitates the utilization of knowledge that a firm receives from external networks and promotes innovation (Fabrizio, 2009; Powell et al., 1996). Therefore, firms with higher absorptive capacity effectively acquire knowledge from their social capital and utilize it for innovation ( Lee et al., 2001) which ultimately enhances the innovative capability of firms (Sheng & Hartmann, 2019). Cohen & Levinthal (1990) suggest that external knowledge sources are important for innovation, but it depends on how capable an organization is to absorb and utilize this knowledge. The social capital of firms empowers the firms to attain knowledge (Fleming & Sorenson, 2004; Nelson, 1982) and firms’ absorptive capacity enables it to value, assimilate and apply for innovation (Arora & Gambardella, 1994; Powell et al., 1996). More explicitly, when firm gets the knowledge from its social capital, the absorptive capacity of the firm impacts its utilization towards innovation (Kogut & Zander, 1992), the higher the absorptive capacity is, the higher the possibility of innovation is (Yu, 2013). Innovation is the combination of possessed and obtained knowledge (Fabrizio, 2009; Nelson, 1982) and a firm with higher absorptive capacity utilizes this acquired knowledge for better innovative capability (Laumann et al., 1978). Therefore, absorptive capacity can become the base for innovative capability as absorptive capacity refers to the ability of firms to apply external knowledge, whereas this innovative capability refers to the ability of firms to utilize knowledge to produce innovative products. Based on these arguments, we assume that when absorptive capacity is higher, firms harvest more value from knowledge which is reserved through social capital. This leads us to another hypothesis:

H9: Absorptive capacity moderates the relationship between social capital and innovative capability.

**Fig. 1:**
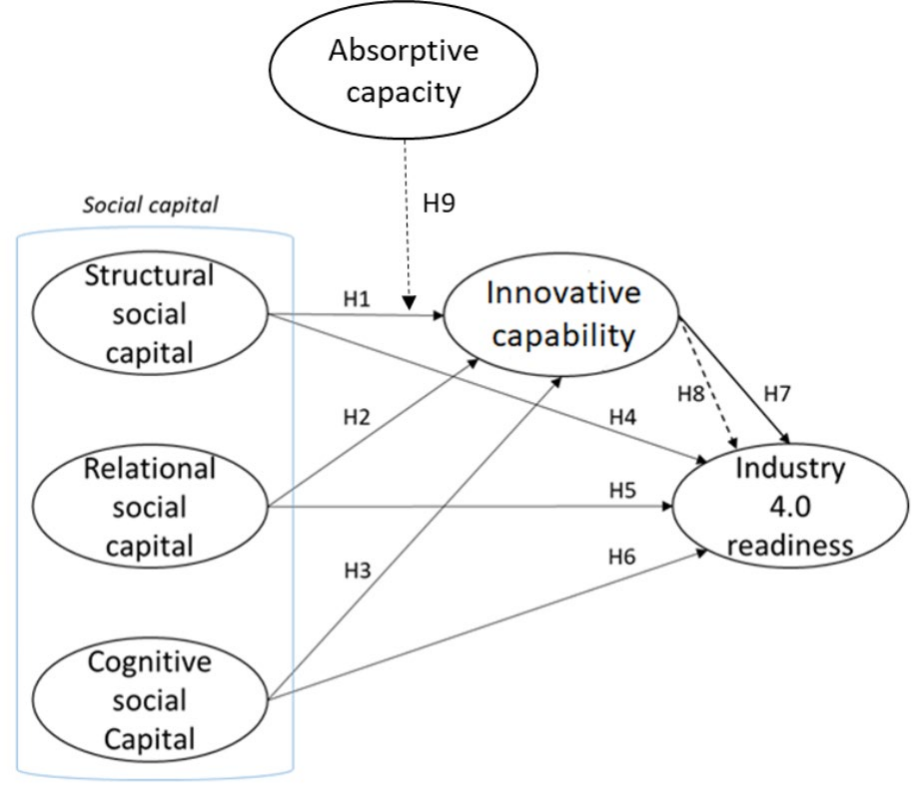
Conceptual model

## Method

### Data collection and sample

The population of this study comprises of manufacturing firms of Pakistan. We chose Pakistan to investigate these issues; particularly Pakistani export firms who have relationship network in technologically advanced economies and extract resources, knowledge, and support from their networks. Pakistan provides a suitable context of developing economy, and it is facing the issue of institutional voids (Khan et al., 2019). Pakistan has started to adopt digital technologies (Nizam et al., 2020) and firms mostly rely on industrialized economies to buy technological products (Malik & Kotabe, 2009). Firms in the developed economies also outsource some of their activities in developed economies e.g. software solutions (Sinkovics et al., 2019). This situation makes social capital an important tool to gain competencies related to Industry 4.0. We collected data from three sources. First, we gathered a list of manufacturing firms in Pakistan using multiple resources such stock exchange, Small and Medium Enterprise Development Authority (SMEDA) and chambers of commerce in different cities. Pakistan stock exchange has three trading floors in three major cities of Pakistan, and it reports 391 large-scale listed manufacturing firms; SMEDA represents Small Scale Manufacturing Companies and there are more than four thousand small-scale firms that are registered with SMEDA. From these sources, we managed to contact senior managers of 534 firms and requested to participate in the online survey. Second, we also requested regional heads of chambers of commerce in difference cities and SMEDA to share out link with firms registered with them. We shared the questionnaire link via email and LinkedIn with all these contacts. In return, we received 320 usable data from 81 firms. Though the response rate is slight low, it is unswerving with existing studies (Shamim et al., 2017, 2020). Also, 320 usable data is sufficient for us to use structural equational model to analyse the hypotheses (Cohen, 1988; Westland, 2010). It is imperative to point out that the unit of analysis for this study is individual firm where questionnaire is filled by multiple employees of top and middle management positions from each firm as they involve with the decision making. The way of collecting data is consistent with existing studies such as Akhtar et al. (2019), Awan et al. (2021), Shamim et al., (2017) and Wamba et al. (2017). Moreover, this measurement method is also consistent with the studies of Eiteneyer et al. (2019) & Mazzucchelli et al. (2019), where cross sectional survey was adopted to investigate relation of all three dimensions of social capital.

### Measures

The constructs of the study are measured using seven-point Likert scales modified from prior literature. It is ranged from 1 (“strongly disagree”) to 7 (“strongly agree”). Three items measuring structural social capital (Cronbach alpha = 0.81) were adapted from Nahapiet & Ghoshal (1998) and Chow & Chan (2008). one example item is “In general, we have a very good relationship with other department.” Four items measuring relational capital (Cronbach alpha = 0.80) were derived from Nahapiet & Ghoshal (1998) and Chow & Chan (2008) measure. One example item of the scale is “We feel connected to our business partners”. Three items measuring cognitive social capital (Cronbach alpha = 0.82) were adapted from (Chow & Chan, 2008) measure. Innovative capability (Cronbach alpha = 0.84) is measured by 4 items adapted from Sheng & Hartmann (2019); five items measuring absorptive capability (Cronbach alpha = 0.84) were adopted from Khan et al. (2019). We used 12 items developed by IBM to measure Industry 4.0 readiness (Cronbach alpha = 0.93). The scale of industry 4.0 readiness is accessible at IBM open source at (https://www.ibm.com/industries/manufacturing/industry-4.0-model-factory/quiz.html).

## Results

Fornell and larcker (1981) approach is followed to test the convergent and discriminant validity. Cronbach alpha is used to examine the reliability of constructs. After carefully weighing the weakness and strength of applying PLS to test hypotheses listed in the literature such as Rönkkö & Evermann (2013); Henseler et al. (2014) and Chin et al. (2003), we decided to adopt PLS in our study. It is difficult to justify the use of PLS for theory testing over SEM (Rönkkö & Evermann 2013). Our rational of using PLS is that PLS considers the measurement model and the structural model simultaneously (Chin et al., 2003). This allows us to test the measurement of variables in the study and the comparably complex relationship between variables including a mediator and a moderator. PLS are used in testing complex model like this study including moderation and mediation effect in addition to have new scales measuring the variables (Ringle et al. 2012). The scale measuring industry 4.0 readiness was developed by IBM, yet it has not been well tested by academic studies. Hence, using PLS would be suitable for this study to test the measurement model whilst test the theoretical model which is consistent with previous studies like Shamim et al. (2017) and Cegarra-Navarro, et al. (2021).

### Sample characteristics

Table 1 presents the characteristics of respondents and their firms. We can see at Table 1 that 91% of firms have over 250 employees with more than 5 million PKR annual sales and nearly 90% of firms are over 5 years old. All the respondents are at managerial level, among which 81.9% are either top managers or executives.

**Table 1:** Sample characteristics

Managerial level	Frequency	%	Age of the firm	Frequency	%
Middle Manager	58	18.1	< 5	8	9.6
Top Manager	196	61.3	6–10	24	29.7
Executive	66	20.6	11–15	21	25.9
**Highest education**			16–20	19	24.1
Secondary school	31	9.7	21–25	5	6.6
Undergraduate	72	22.5	> 25	4	4.4
Graduate	168	52.5	**Number of employees**		
Masters	49	15.3	< 250	7	9.1
**Age of participant**			251–1000	58	71.3
< 30	107	33.4	> 1000	16	19.7
30–35	75	23.4			
36–40	37	11.6	**Annual Sales (PKR)**		
41–45	80	25.0	> 5 Million	14	17.5
> 45	21	6.6	5 Million − 10 Million	41	50.6
**Experience**			> 10 Million	26	31.9
< 5	104	32.5			
6–8	79	24.7			
9–11	72	22.5	N = 81		
12–14	60	18.8			
> 15	5	1.6			

### Reliability and validity

We used confirmatory factor analysis to measure the convergent validity by following Fornell and Larcker (1981) approach which suggests that factor loadings of the constructs should be greater than 0.65, average variance extracted (AVE) and composite reliability (CR) of constructs should be more than 0.50. Results at Appendix 1 indicate that all of these requirements are met. Factor loadings for all the constructs are greater than 0.65, AVE and CR of all the constructs are also greater than minimum required value. These results suggest that convergent validity is established.

To establish discriminant validity, AVE of each construct should be less than squared correlation among constructs (Fornell & Larcker, 1981). Table 2 shows that squared correlations of all the constructs is less than AVE. AVE is mentioned at the diagonal in bold. Mean and standard deviations are also given in Table 2. These results indicate that discriminant validity is established. Chi-square of model is 2236.94, and R-square of outcome variable is 0.70.

**Table 2:** Discriminant validity

Factors		1	2	3	4	5	6
1	Cognitive capital	0.**74**					
2	Innovative capability	0.21	**0.67**				
3	Industry 4.0 readiness	0.45	0.38	**0.58**			
4	Relational capital	0.41	0.26	0.49	**0.63**		
5 6	Structural capital Absorptive capacity	0.17 0.24	0.20 0.56	0.40 0.38	0.34 0.36	**0.73** 0.37	**0.61**

Note: AVE is at the diagonals in bold

### Hypotheses testing

Firstly, we examined the direct association of structural capital, relational capital, and cognitive capital with innovative capability, and Industry 4.0 readiness. Results support the direct association of innovative capability with structural capital (β = 0.20, p < 0.01), relational capital (β = 0.26, p < 0.001), and cognitive capital (β = 0.21, p < 0.001). Based on these findings H1, H2, and H3 are accepted. Furthermore, all three dimensions of social capital are significantly and positively related to Industry 4.0 readiness i.e. structural capital (β = 0.26, p < 0.001), relational capital (β = 0.23, p < 0.001), and cognitive capital (β = 0.30, p < 0.001). It supports H4, H5, and H6. Results also indicate significant indirect relationship of all three dimensions of social capital with Industry 4.0 readiness i.e. structural capital (β = 0.05, p < 0.05), relational capital (β = 0.06, p < 0.01), and cognitive capital (β = 0.05, p < 0.01). Results show that association of innovative capability and Industry 4.0 readiness is significant and positive i.e. (β = 0.24, p < 0.001), it supports H7.

After testing the direct relationship, we then examined the mediation of innovative capability in the relationship of social capital and Industry 4.0 readiness. For mediation analysis we combine the dimension of social capital and transformed it as a single construct of social capital. This method of transformation is consistent with existing literature (Shamim et al., 2017). The mediation of innovative capability in the relationship of social capital and Industry 4.0 readiness is significant i.e. (β = 0.04, p < 0.01), and this mediation is also partial because direct relationship is still significant i.e. (β = 0.66, p < 0.001). These finds support H8. However, results do not support the assumption that absorptive capacity moderates the relationship of social capital and innovative capability, the moderation is not significant, and t-value does not justify the moderation (β = 0.66, p > 0.01). Results are summarized in Table 3.

**Table 3:** Path analysis

Path	Direct effects β/t-value	indirect effects β/t-value	Moderating effect β/t-value	Total effects β/t-value	Hypotheses	Result
Structural social capital ◊ Innovative capability Relational social capital ◊ Innovative capability	0.16**/3.15 0.26***/3.56				H1 H2	Accepted Accepted
Cognitive social capital ◊ Innovative capability	0.21***/3.54				H3	Accepted
Structural social capital ◊ Industry 4.0 readiness	0.26***/6.28	0.05**/2.68		0.32***/6.70	H4	Accepted
Relational social capital ◊ Industry 4.0 readiness	0.23***/4.06	0.06**/2.85		0.29***/5.12	H5	Accepted
Cognitive social capital ◊ Industry 4.0 readiness	0.30***/6.51	0.05**/2.91		0.35***/7.38	H6	Accepted
Innovative capability ◊ Industry 4.0 readiness	0.24***/5.70				H7	Accepted
Social capital ◊ Innovative capability ◊ Industry 4.0 readiness (Social capital*Absorptive capacity) ◊ Innovative capability Age of Firm ◊ Industry 4.0 readiness No of Employees ◊ Industry 4.0 readiness	0.66***/18.36 -0.138*/-2.309 -0.049/-0.826	0.04**/5.03	-0.081(1.9)	0.80***/41.5	H8 H9	Accepted Rejected
*Overall model of R*^2^ = 0.70						

*Note*: ***p < 0.001; ** *p* < 0.01

## Discussion

In the context of developing economy, this study examines the role of firm’s social capital in developed economies in Industry 4.0 readiness. For this purpose, social capital of firms in Pakistan is measured and tested in relation with industry 4.0 readiness. Pakistan provides a suitable context because its firms are at initial stages of adapting Industry 4.0 technologies (Nizam et al., 2020) and mainly relying on importing these technologies from developed economies (Malike & Katobe, 2009). We investigated the mediating role of innovative capability in the relationship of social capital and Industry 4.0 readiness. Furthermore, we also examined the moderation of absorptive capacity in the relationship of social capital and innovative capability. Results suggest that social capital with firms in developed economies is a useful tool to enhance Industry 4.0 readiness in less developed economies such as Pakistan. Furthermore, results of the study also show that innovative capability is positively associated with Industry 4.0 readiness which is consistent with existing literature (Shamim et al., 2016; Sheen & Yang, 2018). Social capital is often discussed as antecedent of innovation and transformation (Maurer et al., 2011), and industry 4.0 readiness can trigger digital transformation in the firms and economy as a whole. Furthermore, this study found that innovative capability mediates the relationship of social capital and Industry 4.0 readiness. In the context of this study it means, firms in developing economy with good social capital in develop economies are in good position to gain innovative capability, which in turn enhances Industry 4.0 readiness. Moreover, firms with strong intra-organizational networks could enhance knowledge sharing, especially for tacit and complex knowledge, and then contribute to innovation (Pérez-Luño et al., 2011).When organizations are capable to adapt to changing environment through continuous innovation, such as the adaptation of the latest technology in the work place and the application of big data at decision-making, then they will be more equipped to embrace industry 4.0 (Shamim, Zeng, et al., 2019). However, results do not support the moderating role of absorptive capacity. One of the reasons for not having significant moderating role of absorptive capacity is that this study measured absorptive capacity as whole instead of separately measuring potential and realized absorptive capacity. Literature suggests that potential absorptive capacity and realized absorptive capacity can have different outcomes (Khan et al., 2019). The former accentuates the significance of acquiring and assimilation of external knowledge, whereas the later elucidates that how firm transforms and exploit this knowledge for competitive advantage (Khan et al., 2019). Moreover, realized capacity also explains the ability of a firm to use acquired knowledge for long run commercial purpose (Sun & Anderson, 2010). Therefore, it might be pertinent to quantify both dimensions of absorptive capacity separately in order to receive more detailed outcome.

### Theoretical Implications

This study contributes towards existing body of knowledge in number of ways. First, it extended the literature of industry 4.0 readiness through the social-technical perspective. This is in line with the recent suggestions made by some scholars who posit that industry 4.0 readiness does not only require organizations to invest on technologies, they should also look inward to improve their internal environment such as managerial support (Agostini and Filippini 2019; Agostini and Nosella, 2020), but also take their external environment in consideration (e.g.Dalenogare, Benitez and Ayala (2018). Our study shows that social capital generated with external business partners can contribute to the organizations’ industry 4.0 readiness in developing countries. Additionally, instead of looking into the direct relationship between social capital to industry 4.0 readiness, we brought innovative capability as a mediator. This adds more explanation to the current understanding of industry 4.0 by showing that social capital could improve organizations’ innovative capabilities which equips organizations to be ready for the new industrial era. Furthermore, the study contributes to understanding industry 4.0 in an emerging economy. As Dalenogare et al. (2018) indicated that the context of the country could have influence of the adoption of technologies. Despite the differences in context, our findings are consistent with Agostini and Nosella (2020)’s findings about the positive impact on industry 4.0 readiness based in European countries. This could mean that strong external social capital could increase organizations’ industry 4.0 readiness regardless of cultural differences.

### Managerial implication

This study offers some managerial implications for firms in less developed economies particularly those in the transition process of digitization and adapting Industry 4.0 technologies. Considering the limitations of institutional support in research and development, firms need to rely more on their social capital developed with firms in industrialized economies. The finding of our study confirms Maurer et al. (2011) that social capital is an effective tool to extract knowledge from external sources using intra-organizational ties to foster innovation. Hence, managers in firms should look outwards to facilitate collaborations with external business partners to develop their social capital. Managers should create opportunities for employees to interact frequently to allow employees to acquire privileged information, resource and knowledge, especially the tacit knowledge which are difficult to codify. Additionally, managers are recommended to develop and maintain trust with their business partners, so the knowledge can be shared without going through formal contracts in business transaction (R. Lee, 2009)(Lee, 2009). Furthermore, to harness cognitive social capital, firms in developing economies should try to agree with foreign firms that what is important, share same ambitions and vision, and should have collective goals. Though all these dimensions are significantly related to Industry 4.0 readiness, however cognitive social capital shows the strongest association. Based on this finding, managers should specially pay attention at developing shared language and value to solidify their relationship. When the business partners have distinctively cultural differences, creating code of conduct can support the quality of communication.

The findings have also emphasized the importance of innovative capability in firms’ industry 4.0 readiness, which is in line with Agostini and Filippini (2019) and Shamim et al. (2016). The interaction with business partners could be valuable resource for firms to gain information and knowledge; managers must utilize the social capital to improve their innovative capability through refining or/and radically change the existing products to meet the market needs. During the process of innovation, if managers can collect data associated with product quality, process flow and adopt relevant emerging technologies in their production system, this will prepare them to be competitive during industry 4.0.

## Conclusions

This study concludes that social capital of firms of developing economies with the developed economies is a useful tool to enhance industry 4.0 readiness. The results of this study indicate that the firms in developing countries with strong social ties in developed countries gain innovative capability which enhances industry 4.0 readiness. Moreover, results also reveal that the firms in developing countries also contribute to innovation through knowledge sharing by using intra-organizational networks with developed economies. Our findings on the mediating relationship of innovative capability show a more direct and indirect relationship between social capital dimensions and industry 4.0 readiness. Overall, we conclude that the important and effective use of social capital in the generation of valuable knowledge and information may shape the relationship between social capital and industry 4.0 readiness. Our findings provide a better understanding each dimension of social capital may enhance industry 4.0 readiness in developing economies.

### Limitations and future research

There are few limitations of this study. This study collected data only from Pakistan which provides a context of developing economy. Future research should collect data from other regions. Another limitation of this study is that we used nested data for our analysis. i.e., responses from managers were nested in firm level data. However, we did not aggregate the responses from each firm and keep multiple responses from single firms. It is mainly because of a smaller number of firms in our sample i.e., 81 which was not appropriate for structural equation modelling. However, our method is consistent with Awan et al. (2021). This study measured absorptive capacity as single construct and results shows that absorptive capacity does not moderate the relationship of social capital and innovative capability. Scholars in the future study could examine the moderating role of potential and realized absorptive capacity separately. It may show different results. Future research can also examine the competitiveness of firm’s social capital using framework of resource-based view and DCs and then test its effect in relation to Industry 4.0. Finally, Covid-19 is a unique context which unprecedentedly challenged many organizations’ readiness at adapting technologies in their work at home within a limited time. Organizations with rich social capital and high absorptive capability may had a smooth transition to a new way of working during and after pandemic as a result of industry 4.0 readiness. This would be an interesting avenue to explore for future studies.

### Electronic Supplementary Material

Below is the link to the electronic supplementary material.

Supplementary Material 1

## Appendix-1: (Questionnaire items)

**Table:**

Variable	Items	Factor loadings	AVE	CR
**Structural capital**	1. In general, we have a very good relationship with other departments 2. Our relationship departments know what knowledge we have at our disposal 3. We know what knowledge could be relevant to which department	0.86 0.84 0.85	0.73	0.89
**Relational capital**	1. We feel connected to our business partners 2. We know our business partners will always try and help us out if we get into difficulties 3. We can trust our business partners to lend us a hand if we need it 4. We can rely on our business partners when we need support in our work	0.79 0.84 0.80 0.73	0.63	0.87
**Cognitive capital**	1. Our business partners and we always agree on what is important at work 2. Our business partners and we always share the same ambitions and vision at work 3. Our business partners and we are always enthusiastic about pursing the collective goals and missions of the whole organization	0.83 0.90 0.84	0.74	0.89
**Social capital**	1. Structural capital 2. Relational capital 3. Cognitive capital	0.82 0.88 0.77	0.69	0.87
**Innovative capability**	1. We frequently refine existing products and services 2. We regularly implement small adaptations to existing products and service 3. We accept demands that go beyond existing products and services 4. We experiment with new products and services in our local market	0.81 0.88 0.81 0.77	0.67	0.89
**Absorptive capacity**	1. My company had the managerial competence to absorb the technology 2. My company had information on the state of the art of the technology 3. My company has a common language to deal with the technology 4. My company has the necessary skills to implement the technology 5. My company has the ability to integrate and apply external knowledge for improving components and processes.	0.73 0.75 0.81 0.83 0.76	0.61	0.88
**Industry 4.0 readiness**	1. We use IoT sensors and AI to enable a proactive approach that supports team and our machines in real time. 2. We are collecting a lot of machine equipment data, but we are not sure how to use it for more than routine operation logistics. 3. Most of our data comes from routine manual data collections. 4. Custom quality assurance models use real-time data feeds to track how critical variables (e.g., temperature, sound, pressure, etc.) impact product quality and process flow. 5. We use generalized benchmarks, and workers on the floor are relied upon to identify and report any major issues. 6. We don’t usually recognize a quality risk until it has been identified down the assembly line. 7. Feedback flows easily within our organization for constant communication – data is also shared with our suppliers, customers and partners. 8. We are working to connect different communication feeds internally and externally, but it’s proving to be a challenge. 9. Our communication isn’t integrated, so information gets stuck in silos. 10. Our organization is developing a 4.0 technical framework, and we’re exploring the role our partners will need to play. 11. My team is just beginning to build a case for Industry 4.0 to put in front of senior leadership. 12. We have a basic understanding of what Industry 4.0 could do for our organization, but we need to learn more.	0.71 0.72 0.67 0.73 0.79 0.81 0.77 0.79 0.81 0.80 0.79 0.69	0.58	0.94
