# Supplementary material for: Inter-organizational social capital of firms in developing economies and industry 4.0 readiness: the role of innovative capability and absorptive capacity
Source: Rev Manag Sci. 2022 Mar 26;17(2):661–82. doi: 10.1007/s11846-022-00539-3 (PMC8959805; doi:10.1007/s11846-022-00539-3)
Supplement: Supplementary file 1 — Supplementary Material 1 [file 11846_2022_539_MOESM1_ESM.docx]

Editors

Review of Managerial Science

17-01-2021

Dear editors,

Please find enclosed the manuscript entitled ‘Interorganizational social capital of firms in developing economies and Industry 4.0 readiness: the role of innovative capability and absorptive capacity’ which we would like to submit for publication in *Review of Managerial Science*. We would be very grateful if you could consider it for review.

We believe that our paper shows a good fit to the journal’s aim, especial it investigates Interorganizational social capital of firms in industry 4.0 readiness. This is a much-needed research area for organizations to understand how to prepare themselves for industry 4.0. This study provides an insight into understand the value of interorganizational social capital in developing economies.

We do hope that our manuscript meets the high standards of the Journal and contributes both to management scholarship and practice. We declare that the submitted paper has not been previously published nor is currently under consideration for publication elsewhere.

We look forward to hearing from you.

Kind regards,

The Authors
